# Epidemiological and functional insights into *iroBCDN* loss in ST11 carbapenem-resistant hypervirulent *Klebsiella pneumoniae*

**DOI:** 10.1128/spectrum.02479-25

**Published:** 2025-12-15

**Authors:** Jiawei Ding, Muli Xu, Mengying Zhang, Jiyong Chang, Zidan Hu, Yan Yu, Yao Yao, Ni Shen, Wenlin Tai, Lei Feng

**Affiliations:** 1 Department of Medical Laboratory, The Affiliated Yan'an Hospital of Kunming Medical University 619366 , Kunming, Yunnan, People’s Republic of China; 2 Department of Blood Transfusion, The Affiliated Yan'an Hospital of Kunming Medical University 619366 , Kunming, Yunnan, People’s Republic of China; 3 Department of Medical Laboratory, The Second Affiliated Hospital of Kunming Medical University 66472 , Kunming, Yunnan, People’s Republic of China; Cinvestav-IPN, Mexico City, Mexico

**Keywords:** carbapenem-resistant *Klebsiella pneumoniae*, hypervirulence, ST11, *iroBCDN*, fitness

## Abstract

Carbapenem-resistant hypervirulent *Klebsiella pneumoniae* (CR-hvKP) has emerged as a major public health threat due to its high virulence, multidrug resistance, and increasing global prevalence. However, the molecular characteristics and adaptive mechanisms underlying the CR-hvKP pathogenesis remain poorly understood. In this study, we collected 217 CRKP isolates from a tertiary hospital and identified 46 as CR-hvKP. Genomic analysis revealed that the majority of CR-hvKP strains belonged to ST11-KL64 (48.7%, 20/46) and ST11-KL25 (46.3%, 19/46), followed by ST11-KL47 (4.8%, 2/46). Interestingly, while *iroBCDN* was present in various sequence types, it was found in ST11 strains only with the KL47 capsule type, which showed high virulence in *Galleria mellonella* models. To investigate the biological role of *iroBCDN*, we constructed an *iroBCDN* deletion mutant and a complemented strain in an ST11-KL47 background. The lactate dehydrogenase cytotoxicity assays and *G. mellonella* infection models revealed no significant difference in virulence among the wild-type, knockout, and complemented strains. Remarkably, phenotypic assays showed that the *iroBCDN* deletion mutant exhibited enhanced growth fitness, competitive advantage, oxidative stress resistance, and survival in human whole blood. Transcriptomic analysis revealed that *iroBCDN* deletion led to the upregulation of genes involved in oxidative stress response, capsule biosynthesis, and energy metabolism, while genes related to fimbrial assembly and carbon metabolism were downregulated. These findings suggest that the loss of *iroBCDN* does not attenuate virulence in ST11 CR-hvKP but instead enhances its environmental adaptability, potentially contributing to the persistence and dissemination of epidemic clones.

**IMPORTANCE:**

The emergence of carbapenem-resistant hypervirulent *Klebsiella pneumoniae* (CR-hvKP) poses a severe global health threat. This study reveals a critical paradox: deletion of the *iroBCDN* locus traditionally associated with virulence does not attenuate pathogenicity in ST11-KL47 CR-hvKP. Instead, its loss significantly enhances bacterial fitness by improving growth competitiveness, oxidative stress resistance, and survival in human blood. It demonstrates how loss of specific genetic elements may facilitate the dominance of high-risk clones like ST11-KL64/KL25 by optimizing environmental adaptation and persistence—key factors in hospital transmission. Understanding this fitness trade-off is vital for developing strategies against resilient CR-hvKP epidemics.

## INTRODUCTION

*Klebsiella pneumoniae* is a gram-negative opportunistic pathogen that can cause a wide range of community-acquired and hospital-associated infections, such as pneumonia, sepsis, urinary tract infections, and brain and liver abscesses (1). Based on differences in virulence potential, *K. pneumoniae* strains are generally classified into two groups: classical *K. pneumoniae* (cKp) and hypervirulent *K. pneumoniae* (hvKp) (2). hvKp has attracted increasing attention due to its ability to cause severe and invasive infections, including pyogenic liver abscess, endophthalmitis, and meningitis (3–5). Carbapenem resistance has become increasingly prevalent due to the widespread application of carbapenem antibiotics and invasive medical procedures (6). Through horizontal gene transfer, strains may acquire both resistance and virulence determinants, leading to carbapenem-resistant hypervirulent *K. pneumoniae* (CR-hvKP), which has emerged as a serious public health concern (7).

CR-HvKP is increasingly prevalent in healthcare settings, with sequence type 11 (ST11) being one of its most dominant high-risk lineages (8). ST11 strains exhibit strong environmental adaptability, high levels of multidrug resistance, including resistance to carbapenems, and enhanced virulence (9). Since the first report of CR-hvKP in China in 2015, ST11 CR-hvKP has rapidly spread and caused multiple outbreaks (10). A notable outbreak occurred in 2018 in an intensive care unit in China, resulting in multiple deaths (11). ST11 CR-hvKP has subsequently become the most prevalent clone in China, highlighting the urgent need to understand its transmission and colonization mechanisms (12). The virulence of hvKp is associated with a variety of factors, including capsular polysaccharides, siderophore-mediated iron acquisition systems, lipopolysaccharides, pili, and virulence plasmids (13). Among these, iron acquisition systems and capsular polysaccharides are considered to be the most critical determinants of pathogenicity (14). Iron is essential for bacterial metabolism, growth, and survival, especially within the iron-limited environment of the host (15). The *iroBCDN* gene cluster encodes salmochelin, a catecholate-type siderophore that allows the bacterium to evade host immunity by bypassing lipocalin-2 binding (16). Previous studies have demonstrated that *K. pneumoniae* strains producing salmochelin display increased virulence and enhanced colonization in the respiratory tract of animal models (17). Moreover, salmochelin production has been strongly associated with the development of sepsis (16).

The *iroBCDN* gene cluster is typically located on a pLVPK-like virulence plasmid, along with other key virulence genes, such as *iucABCD-iutA* and *rmpA*/*rmpA2* (18). Acquisition of this plasmid by CRKP can result in the formation of CR-hvKP strains with both resistance and virulence characteristics (19). However, recent genomic analyses have revealed that the *iroBCDN* gene cluster is frequently deleted in ST11 CR-hvKP isolates. Notably, the absence of this virulence factor does not appear to reduce the epidemic potential of these strains (20). Although the high prevalence of *iroBCDN* deletion in ST11 CR-hvKP strains has been documented, the underlying molecular mechanisms that contribute to this adaptive advantage remain poorly understood. It is hypothesized that the loss of this gene cluster may alter the physiological state and stress response pathways of the bacterium, thereby promoting its survival and dissemination in clinical settings.

This study characterizes ST11 CR-hvKP and explores the impact of *iroBCDN* deletion on bacterial physiology, virulence, and adaptability. Through integrated genomic and phenotypic analyses, we show that locus loss alters fitness traits that favor persistence and spread in clinical settings.

## MATERIALS AND METHODS

### Bacteria collection and identification

A total of 217 non-duplicate carbapenem-resistant *Klebsiella pneumoniae* (CRKP) isolates were collected from clinical specimens at the Yan'an Hospital of Kunming City, China between 2021 and 2024. Bacterial identification was initially performed using matrix-assisted laser desorption/ionization time-of-flight mass spectrometry (MALDI-TOF MS) (Bruker Daltonics, Germany) and analyzed with the Bruker Biotyper software (version 3.0). The identification score values for all isolates are presented in Table S1. The minimum inhibitory concentrations (MICs) of these carbapenems (meropenem, imipenem, or ertapenem) were determined for all isolates by the broth microdilution method following CLSI M100-34 guidelines, with each test performed in triplicate. CRKP was defined as resistance to at least one carbapenem antibiotic. To identify hypervirulent strains, the presence of virulence-associated genes (*rmpA*, *rmpA2*, *iucA*, *peg-344*, and *iroB*) was evaluated by PCR using primers designed based on previously published sequences (21). Based on this criterion, 46 CRKP isolates were categorized as carbapenem-resistant hypervirulent *K. pneumoniae* (CR-hvKP), and the MIC results are summarized in Table S2. Two reference strains, NTUH-K2044 (a hypervirulent reference strain) and ATCC 700603 (a low-virulence reference strain), were included as positive and negative controls, respectively, and maintained in our laboratory for comparative analyses.

### Whole-genome sequencing and bioinformatic analysis

Genomic DNA was extracted from *K. pneumoniae* isolates using the TIANamp Bacteria DNA Kit (Tiangen, China) following the manufacturer’s instructions. Whole-genome sequencing was performed on the Illumina NovaSeq 6000 platform, generating 150 bp paired-end reads. Raw reads were quality-checked using FastQC (https://www.bioinformatics.babraham.ac.uk/projects/fastqc/) and trimmed with Trimmomatic to remove low-quality bases and adapter sequences (22). Clean reads were *de novo* assembled using SPAdes v3.14.1 (23). Multilocus sequence typing (MLST) was performed using the mlst tool (https://github.com/tseemann/mlst). The capsule (K) and lipopolysaccharide (O) locus types were identified using Kaptive (24). Antimicrobial resistance genes and virulence factors were detected with ABRicate using the ResFinder and VFDB databases, respectively. Resistance and virulence scores were calculated using Kleborate v3 (25), and detailed scores for all isolates are presented in Table S2. Core-genome SNPs were identified using Snippy v4.6.0 (https://github.com/tseemann/snippy), with *K. pneumoniae* HS11286 (RefSeq: GCF_000240185.1) as the reference genome. A maximum-likelihood phylogenetic tree was constructed using IQ-TREE v2.2.6 (26), and the final tree was visualized and annotated with Interactive Tree of Life (iTOL ) (27).

### Construction of *iroBCDN* deletion mutant

The *iroBCDN* deletion mutant was constructed using a CRISPR-Cas9-mediated genome editing system (28). Primers used in this study were listed in Table S3. Two plasmids, pCasKP-apr (encoding Cas9 and λ-Red recombinase) and pSGKP-Hyg (carrying the single-guide RNA and homologous donor DNA), were used in combination. Briefly, the donor DNA fragment containing ~500 bp homologous arms flanking the *iroBCDN* locus was amplified by PCR and cloned into pSGKP-Hyg, which also carried a designed spacer targeting the *iroBCDN* gene cluster. The recombinant pSGKP-Hyg plasmid and the linear donor DNA were co-electroporated into *K. pneumoniae* cells harboring pCasKP-apr after induction with L-arabinose. Transformants with the desired deletion were selected on LB agar plates supplemented with 50 mg/L hygromycin B and 5% (w/v) sucrose and incubated at 30°C to facilitate plasmid curing. Colonies were screened by colony PCR using primers flanking the *iroBCDN* region, and the deletion was further confirmed by Sanger sequencing. A complemented strain was generated by cloning the intact *iroBCDN* operon with its native promoter into a pDK6 plasmid and introducing it into the deletion mutant by electroporation.

### Cytotoxicity test

The cytotoxicity of *Klebsiella pneumoniae* wild-type and Δ*iroBCDN* mutant strains was assessed using a lactate dehydrogenase (LDH) release assay. Human alveolar epithelial A549 cells were seeded in 24-well plates at a density of 1 × 10⁵ cells per well and incubated at 37 °C with 5% CO₂ overnight to allow adherence. Bacterial cultures in the mid-log phase were harvested, washed with phosphate-buffered saline (PBS), and adjusted to the desired concentration. Cells were infected at a multiplicity of infection (MOI) of 100:1 and incubated for 4 h. After incubation, the culture supernatants were collected and centrifuged to remove bacteria and debris. The amount of LDH released into the supernatant was quantified using the LDH Cytotoxicity Assay Kit (Beyotime Biotechnology, China; Cat. No. C0017) according to the manufacturer’s instructions. Absorbance was measured at 490 nm using a microplate reader. All assays were performed in biological triplicates.

### *Galleria mellonella* infection model

The *Galleria mellonella* infection model was employed to evaluate the virulence of *Klebsiella pneumoniae* strains, including the wild-type ST11 CR-hvKP isolate, the Δ*iroBCDN* mutant, and reference strains NTUH-K2044 (high virulence) and ATCC 700603 (low virulence). Larvae weighing 200–250 mg were randomly selected. Larvae were kept at room temperature in the dark without feeding prior to use. Bacterial strains were cultured overnight in LB broth at 37°C with shaking, and then harvested by centrifugation. The pellets were washed and resuspended in sterile phosphate-buffered saline (PBS), and the bacterial concentration was adjusted to 1 × 10⁶ CFU/mL. Each larva was injected with 10 µL of the bacterial suspension into the left posterior proleg using a Hamilton syringe. The negative control group received 10 µL of sterile PBS. Each experimental group consisted of 10 larvae. Following injection, larvae were incubated in Petri dishes at 37°C in the dark. Survival was recorded every 12 h for a total of 72 h. Larvae were considered dead when they showed no movement in response to gentle touch and exhibited complete melanization.

### RNA-Seq and data analysis

Total RNA was extracted from mid-log phase cultures of the wild-type and Δ*iroBCDN Klebsiella pneumoniae* strains using the RNeasy Mini Kit (Qiagen, Germany) following the manufacturer’s instructions. Residual genomic DNA was removed by on-column treatment with RNase-free DNase I. RNA quality and integrity were assessed using the Agilent Bioanalyzer 2100 System (Agilent Technologies, USA). Ribosomal RNA was depleted using the Ribo-Zero rRNA Removal Kit (Illumina, USA), and strand-specific cDNA libraries were constructed using the NEBNext Ultra II Directional RNA Library Prep Kit (New England Biolabs, USA). Libraries were sequenced on an Illumina NovaSeq platform generating 150 bp paired-end reads. Raw sequencing data were assessed using FastQC (https://www.bioinformatics.babraham.ac.uk/projects/fastqc/), and adapters and low-quality reads were trimmed using Trimmomatic (22). Clean reads were aligned to the *K. pneumoniae* NUTH-K2044 reference genome (National Center for Biotechnology Information accession: GCF_000009885.1) using HISAT2 (29). NTUH-K2044 (ST23) was chosen because it carries a complete *iroBCDN* cluster, which enabled accurate annotation and comparison of this locus. Gene-level read counts were obtained using featureCounts, and differential expression analysis was performed using the DESeq package in R. *P*-values were adjusted using the Benjamini-Hochberg procedure for multiple testing. Genes were considered differentially expressed if they exhibited an adjusted *P*-value < 0.05 and |log₂(fold change)| ≥ 1. The functional enrichment analysis of differentially expressed genes (DEGs) was conducted using the Kyoto Encyclopedia of Genes and Genomes (KEGG) PATHWAY database via custom R scripts. The Gene Ontology (GO) enrichment analysis was performed with Goatools.

### Growth curve measurements

To evaluate the impact of *iroBCDN* deletion on bacterial growth, growth curves were generated for the wild-type ST11 CR-hvKP strain and its isogenic Δ*iroBCDN* mutant. Individual colonies of each strain were inoculated into LB broth and incubated overnight at 37°C with shaking at 220 rpm. The bacterial suspensions were adjusted to a final concentration of 1 × 10⁶ CFU/mL. Subsequently, 200 µL of each suspension was transferred into individual wells of a sterile 96-well flat-bottom microplate (Corning, USA). LB broth alone was used as a blank control. The microplate was incubated at 37°C with continuous shaking at 220 rpm. The optical density at 600 nm (OD₆₀₀) was measured every 30 min for a total of 24 h using a microplate reader (BioTek Synergy H1, USA). Each strain was tested in triplicate. Growth curves were plotted based on the average OD₆₀₀ values at each time point. All experiments were independently repeated at least three times to ensure reproducibility and statistical reliability.

### Competition assay

Competition experiments were conducted to evaluate the relative fitness of the wild-type and Δ*iroBCDN K. pneumoniae* strains *in vitro*. Overnight cultures of both strains were adjusted to the same optical density (OD_600_ = 1.0) and mixed at a 1:1 ratio. The mixed cultures were inoculated into fresh Mueller-Hinton (MH) broth and incubated at 37°C with shaking. At 0, 4, 8, 16, and 24 h post-inoculation, 100 µL of each culture was serially diluted and plated on MH agar plates. To distinguish between the wild-type and mutant strains, 200 individual colonies were randomly picked at each time point and subjected to colony PCR using primers targeting the *iroBCDN* gene cluster. Strains were identified based on the presence or absence of *iroBCDN*. Relative fitness (RF) of the Δ*iroBCDN* mutant was calculated using the formula: RF = log₁₀(N24/N0) ÷ log₁₀(M24/M0), where N and M represent the CFU counts of the mutant and wild-type strains, respectively. An RF value less than 1 indicates a fitness disadvantage of the mutant strain relative to the wild type. All experiments were performed in triplicate to ensure reproducibility.

### Biofilm formation assays

Biofilm formation by *K. pneumoniae* wild-type and Δ*iroBCDN* mutant strains was assessed using the crystal violet staining method in 96-well polystyrene microtiter plates. Overnight bacterial cultures were diluted 1:100 in fresh LB broth, and 200 µL of the diluted suspension was added to each well of a sterile 96-well flat-bottom polystyrene plate (Corning, USA). Plates were incubated statically at 37°C for 48 h to allow biofilm formation. After incubation, the planktonic cells were discarded, and the wells were gently washed once with distilled water to remove non-adherent bacteria. The remaining biofilm was fixed with 200 µL of 99% methanol for 15 min, and then air-dried. The wells were then stained with 200 µL of 0.1% (w/v) crystal violet solution (Solarbio, China) for 10 min at room temperature. Excess dye was removed, and the wells were washed three times with distilled water. Crystal violet bound to the biofilm was solubilized with 200 µL of 95% ethanol, and the optical density (OD) was measured at 600 nm using a microplate reader. Each experiment was performed in triplicate and repeated at least three times independently. Data are expressed as mean ± standard deviation (SD).

### Human whole-blood killing assay

Bacterial strains, including the wild-type KP52, Δ*iroBCDN*, and reference strains NTUH-K2044 and ATCC 700603, were cultured in LB broth at 37°C until reaching the logarithmic growth phase. Cultures were then washed twice and resuspended in sterile phosphate-buffered saline (PBS) to a final concentration of 1 × 10⁶ CFU/mL. For each reaction, 25 µL of the bacterial suspension was mixed with 75 µL of freshly collected heparinized whole blood from healthy adult volunteers in sterile 1.5 mL Eppendorf tubes. The mixtures were incubated at 37°C with gentle agitation. At 1, 2, and 3 h post-incubation, 10-fold serial dilutions were prepared in PBS and plated onto LB agar to determine viable bacterial counts (CFU/mL). Bacterial survival was calculated as a percentage of the initial inoculum. Each experiment was performed in triplicate to ensure reproducibility.

### Hydrogen peroxide killing assay

Bacterial strains, including the wild-type KP52, the Δ*iroBCDN*, and reference strains NTUH-K2044 and ATCC 700603, were cultured in LB broth at 37°C to the logarithmic growth phase. Cultures were washed twice and resuspended in sterile phosphate-buffered saline (PBS) to a final concentration of 1 × 10⁶ CFU/mL. For each reaction, 25 µL of the bacterial suspension was mixed with 75 µL of PBS containing hydrogen peroxide (final concentration 3 mM) in sterile 1.5 mL tubes. Mixtures were incubated at 37°C with gentle agitation for 1 h, then serially diluted and plated on LB agar for CFU enumeration. Bacterial survival was calculated as survival rate (%). All assays were performed in triplicate.

### Statistical analysis

All statistical analyses were performed using GraphPad Prism 8 software (GraphPad Software, Inc., San Diego, CA, USA). For comparisons between two groups, a paired *t*-test was used. For comparisons among three or more groups, one-way analysis of variance (ANOVA) was conducted, followed by appropriate post hoc tests when necessary. A *P*-value ≤ 0.05 was considered statistically significant.

## RESULTS

### Clinical characteristics and phylogenetic analysis of 46 CR-hvKP strains

**Fig 1:**
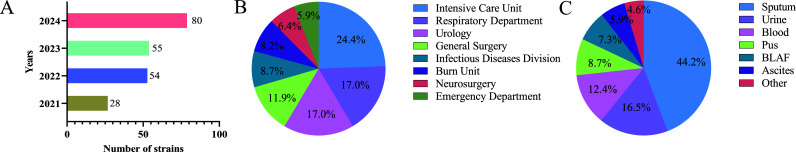
Epidemiological characteristics of the 217 CR-KP clinical isolates collected from 2021 to 2024. (**A**) Annual distribution of CRKP isolates and CR-hvKP detection rates. (**B**) Departmental sources of CRKP isolates. (**C**) Specimen types of CRKP isolates.

**Fig 2:**
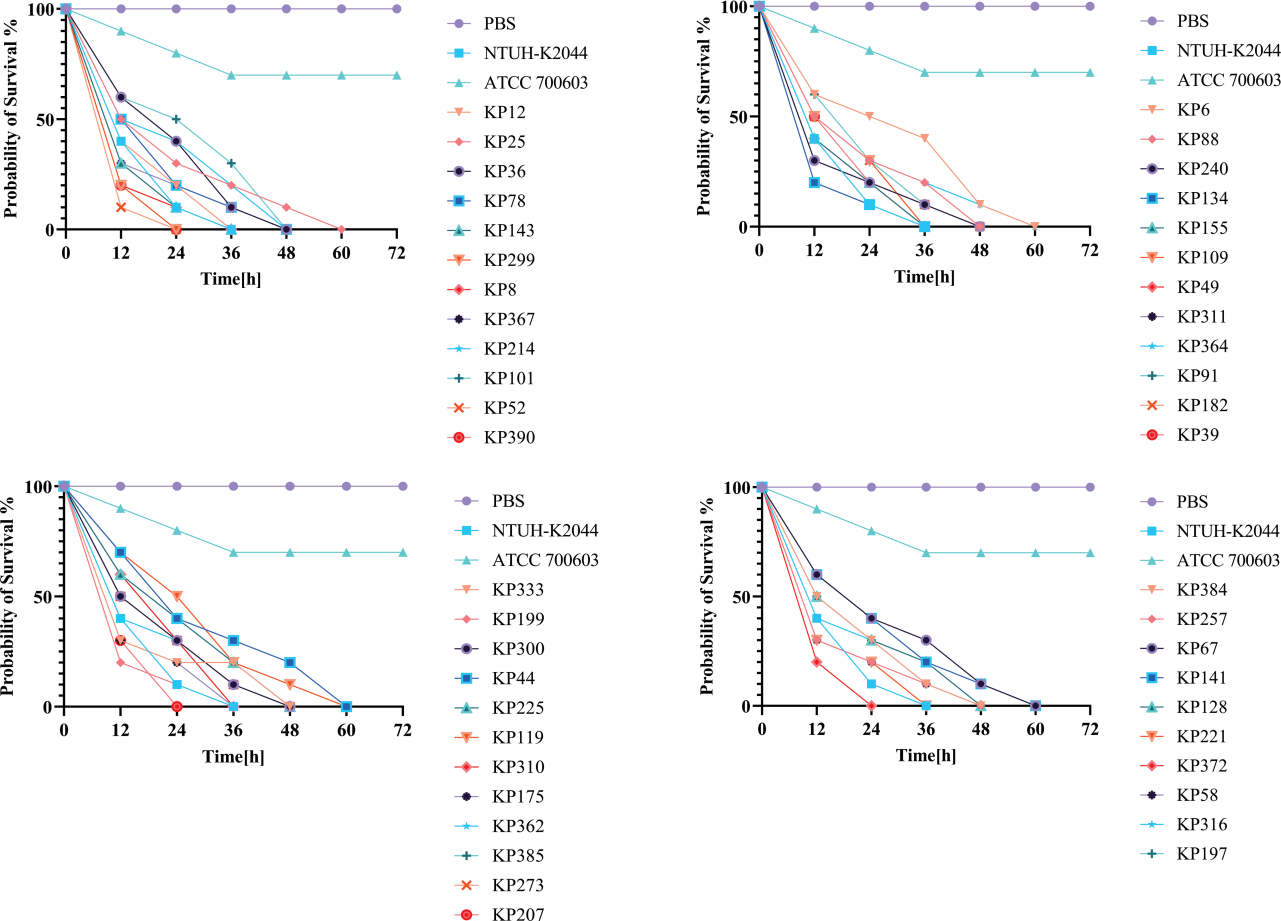
Virulence assessment of the 46 CR-hvKP clinical isolates using the *Galleria mellonella* infection model. Survival curves of the larvae infected with CR-hvKP strains are shown in comparison with the high-virulence control strain NTUH-K2044, low-virulence control strain ATCC 700603, and PBS control. Statistical significance was determined by the log-rank (Mantel-Cox) test.

Between 2021 and 2024, a total of 217 CR-KP clinical isolates were collected. The annual detection rates of CR-hvKP increased over time, with 12.9% (28/217) in 2021, 24.8% (54/217) in 2022, 25.3% (55/217) in 2023, and 36.8% (80/217) in 2024 (Fig. 1A). The majority of isolates originated from the Intensive Care Unit (24.4%, 53/217), Respiratory Department (17.0%, 37/217), and Urology Department (17.0%, 37/217) (Fig. 1B). The most common specimen types were sputum (44.2%, 96/217), urine (16.5%, 36/217), and blood (12.4%, 27/217) (Fig. 1C). Virulence genes (*rmpA*, *rmpA2*, *iucA*, *peg-344*, and *iroB*) were detected via PCR, and isolates carrying at least one of these genes were defined as CR-hvKP. A total of 46 CR-hvKP isolates were identified. The annual distribution of these isolates was relatively balanced: 10 isolates (21.7%) were collected in 2021 and 2022, 12 isolates (26.0%) in 2023, and 14 isolates (30.4%) in 2024. Most isolates were obtained from the Intensive Care Unit (41.3%, 19/46), followed by the Respiratory Department (26.0%, 12/46) and the Emergency Department (10.8%, 5/46). The primary specimen types were sputum (63.0%, 29/46), pus (10.8%, 5/46), and urine (10.5%, 5/46). All 46 strains exhibited significant virulence in the *Galleria mellonella* infection model (Fig. 2). Their survival curves were located between those of the high-virulence control strain NTUH-K2044 and the low-virulence strain ATCC 700603 and were clearly distinct from the PBS control.

**Fig 3:**
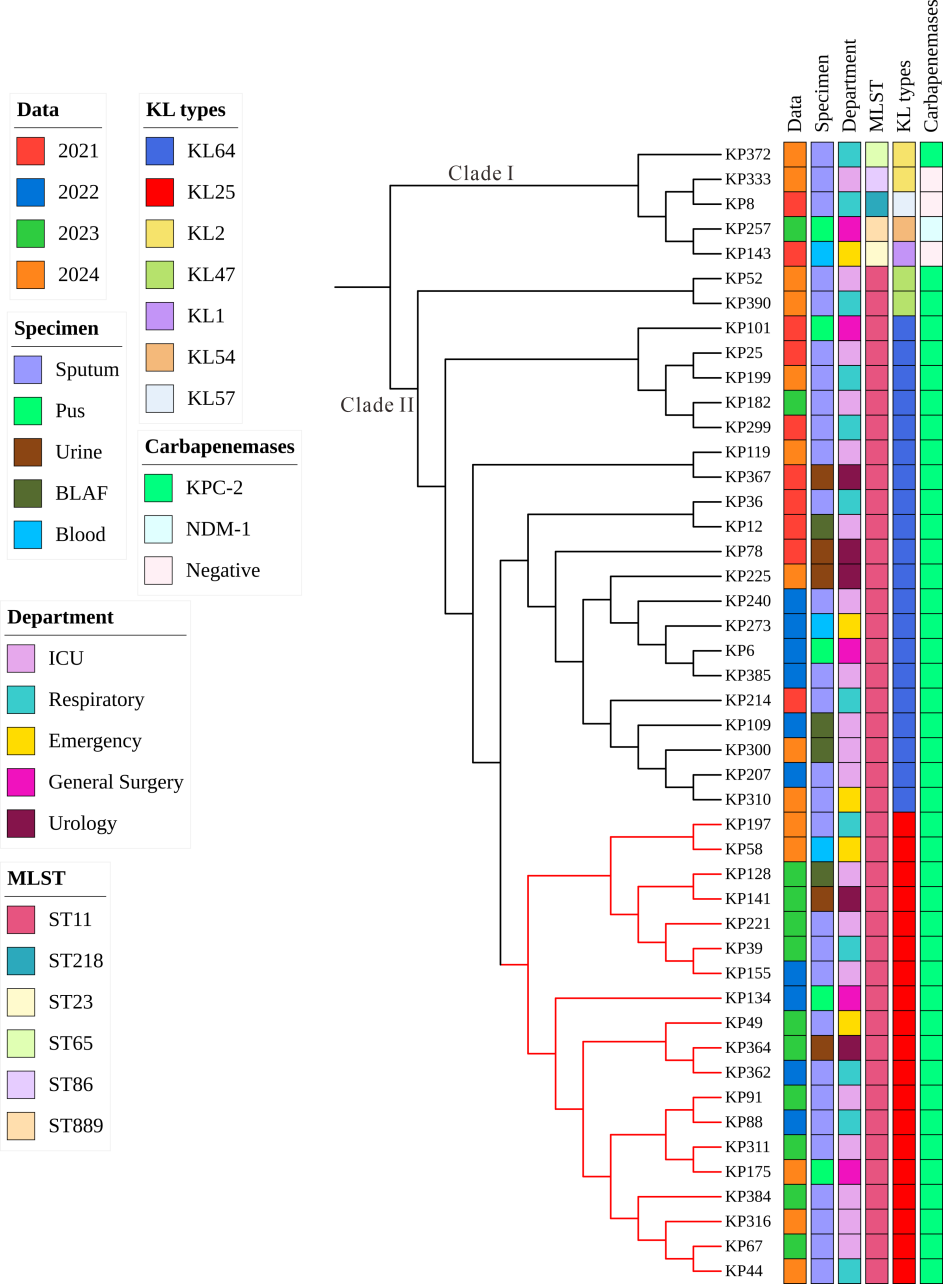
Core-genome phylogenetic analysis of the 46 CR-hvKP clinical isolates. The tree reveals two major clades (Clades I and II), with a subgroup highlighted in red indicating a distinct evolutionary lineage. Metadata associated with each isolate are visualized alongside the tree, including year of isolation (Data), specimen type, hospital department, sequence type (MLST), capsular serotype (KL type), and carbapenemase genotype.

To investigate the genetic characteristics of CR-hvKP, whole-genome sequencing and core-genome phylogenetic analysis were performed on the 46 CR-hvKP isolates (Fig. 3). Multilocus sequence typing (MLST) revealed six distinct sequence types (STs) among the 46 CR-hvKP strains, with ST11 being predominant (89.1%, 41/46). The remaining five isolates belonged to ST218, ST23, ST65, ST86, and ST889 (each 2.1%). Capsule locus (KL) typing showed that within ST11 isolates, KL64 (48.7%, 20/41) and KL25 (46.3%, 19/41) were the most common capsular types, followed by KL47 (4.8%, 2/41). Core-genome phylogenetic analysis revealed two major clades (Fig. 3). Clade I consisted of non-ST11 isolates, including ST218, ST23, ST65, ST86, and ST889. Clade II was composed exclusively of ST11 isolates, which further clustered into distinct sublineages based on capsular types. Notably, KL25 isolates formed a highly monophyletic branch within Clade II (highlighted in red), suggesting recent clonal expansion. This KL25 cluster was phylogenetically close to a sublineage of KL64 isolates, indicating that ST11-KL25 strains likely evolved from ST11-KL64 ancestors through capsular switching events.

### Analysis of antibiotic resistance and virulence genes in 46 CR-hvKP strains

**Fig 4:**
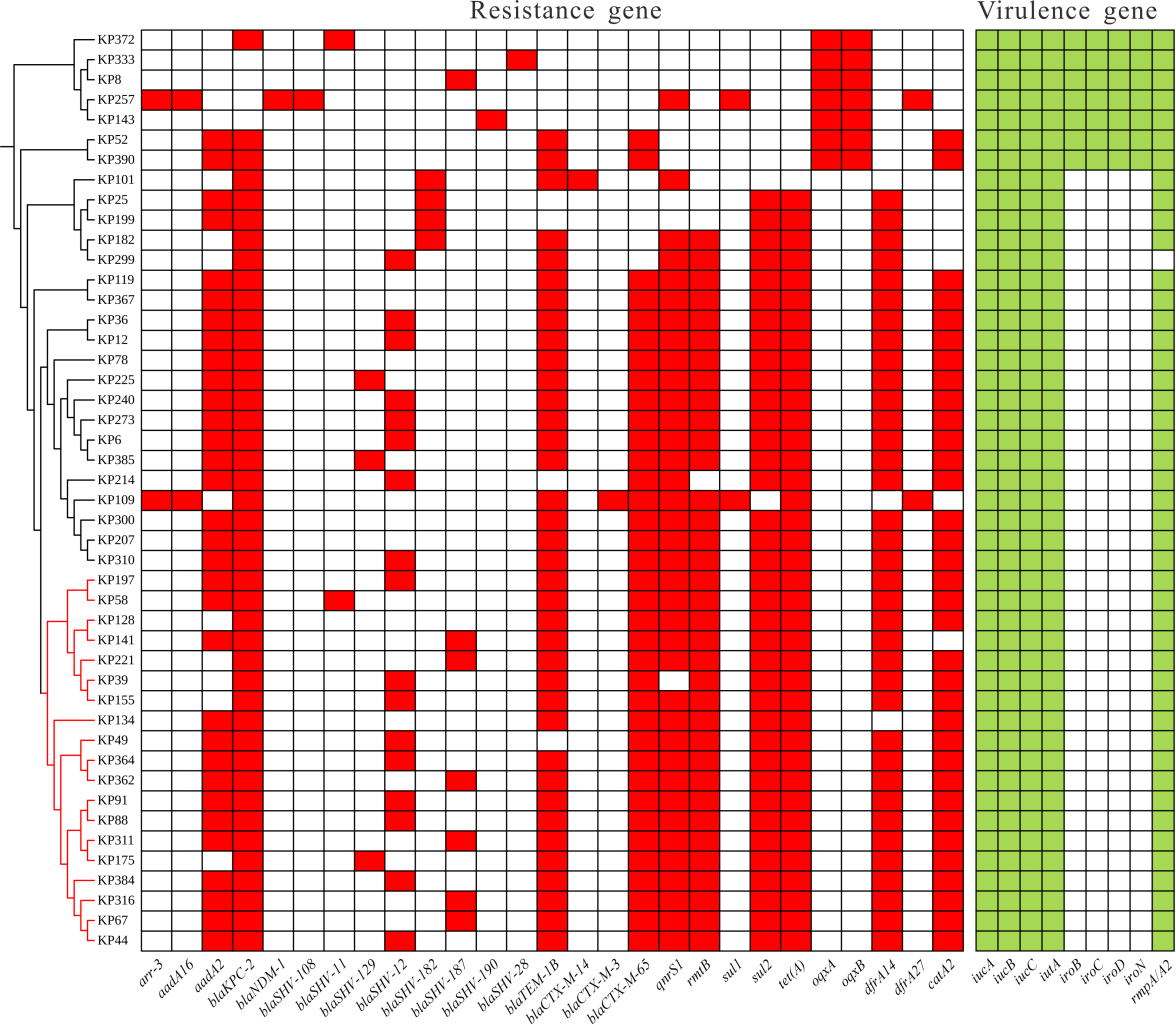
Distribution of the major antimicrobial resistance genes and virulence gene profiles of the 46 CR-hvKP clinical isolates.

Whole-genome analysis of the 46 CR-hvKP isolates revealed a high prevalence of antimicrobial resistance genes. The detection rates were as follows: *arr-3* (4.3%, 2/46), *aadA16* (69.5%, 32/46), *bla*_KPC-2_ (91.3%, 42/46), *bla*_NDM-1_ (2.1%, 1/46), *bla*_SHV_ (78.2%, 36/46), *bla*_TEM_ (80.4%, 37/46), *bla*_CTX_ (80.4%, 37/46), *qnrS1* (80.4%, 37/46), *rmtB* (76.0%, 35/46), *sul* genes (84.7%, 39/46), *tetA* (82.6%, 38/46), *oqx* (15.2%, 7/46), *dfrA* (82.6%, 38/46), and *catA2* (73.9%, 34/46) (Fig. 4). Notably, *bla*_KPC-2_ was the predominant carbapenemase gene, while *bla*_NDM-1_ was detected in only one isolate. Three isolates (KP143, KP8, and KP333) lacked carbapenemase genes but still exhibited carbapenem resistance (Table S2). Further sequence analysis demonstrated that all three lacked intact OmpK35 and OmpK36 and carried ESBL-encoding *bla*_SHV_ variants (*bla*_SHV-190_, *bla*_SHV-187_, and *bla*_SHV-28_, respectively). Virulence gene profiling indicated that all isolates (100%) harbored the aerobactin synthesis cluster (*iutA-iucABCD*) and *rmpA2*, whereas the salmochelin operon (*iroBCDN*) was identified in only 15.2% (7/46) of strains (Fig. 4). Among the 41 ST11 CR-hvKP isolates, only two ST11-KL47 strains carried the *iroBCDN* cluster; none of the ST11-KL64 or ST11-KL25 strains possessed this gene cluster. Kleborate analysis showed that 43 isolates (93.5%) had a resistance score of ≥2, indicating a high level of multidrug resistance. Additionally, 45 isolates (97.9%) had a virulence score ≥ 4.

### The deletion of *iroBCDN* does not reduce the virulence of ST11 CR-hvKP

**Fig 5:**
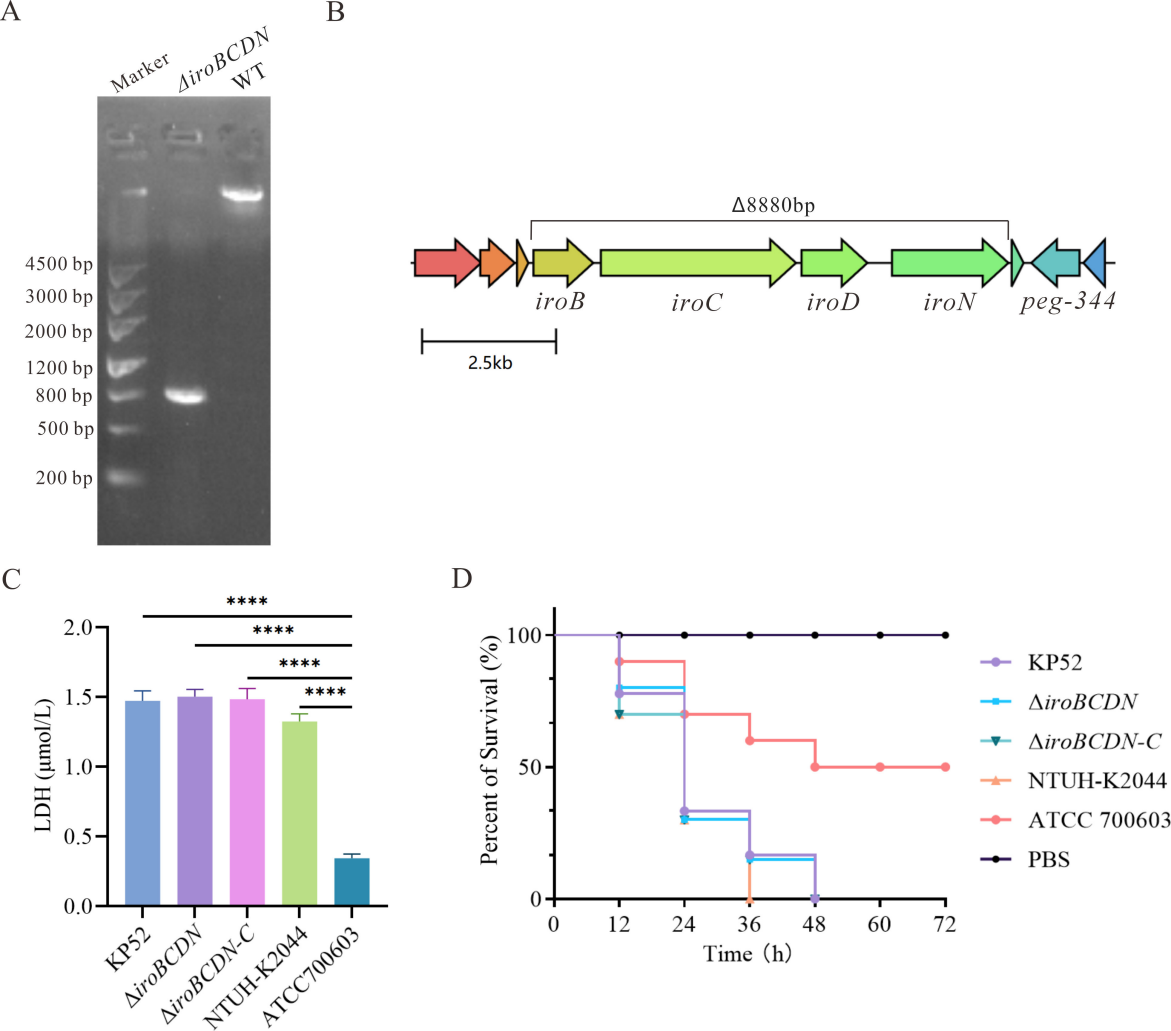
Deletion of *iroBCDN* does not attenuate the virulence of ST11 CR-hvKP. (**A**) PCR verification of *iroBCDN* deletion in strain KP52. (**B**) Construction of the *iroBCDN* deletion mutant. (**C**) Cytotoxicity of KP52, Δ*iroBCDN*, and Δ*iroBCDN*-C assessed by LDH release in A549 cells. Statistical significance was determined by one-way ANOVA with multiple comparisons; ****P < 0.0001. Error bars represent the standard deviation from three independent experiments. (**D**) Survival curves of *Galleria mellonella* larvae. Statistical significance was determined by the log-rank (Mantel-Cox) test. There was no statistically significant difference in survival between KP52 and Δ*iroBCDN*.

To evaluate the contribution of the *iroBCDN* gene cluster to the virulence of ST11 CR-hvKP, an *iroBCDN*-positive ST11-KL47 strain (KP52) was selected for gene knockout. The resulting mutant strain (Δ*iroBCDN*) and its complemented derivative (Δ*iroBCDN-C*) were successfully constructed. The PCR analysis confirmed the deletion of the *iroBCDN* locus, with the fragment from the mutant strain being approximately 8,800 bp shorter than that of the wild-type strain (Fig. 5A and B). To assess virulence, both cytotoxicity and *Galleria mellonella* infection assays were performed. In the LDH release assay, the wild-type strain KP52, Δ*iroBCDN*, and Δ*iroBCDN-C* exhibited comparable cytotoxicity levels, with mean LDH concentrations of 1.47  ±  0.07, 1.50  ±  0.05, and 1.49  ±  0.08 µmol/L, respectively (Fig. 5C). There were no statistically significant differences among the three strains (*P* > 0.05). In contrast, the high-virulence control strain NTUH-K2044 showed a slightly lower LDH release (1.33 ± 0.05 µmol/L), while the low-virulence strain ATCC 700603 exhibited markedly reduced cytotoxicity (0.35 ± 0.03 µmol/L) with a significant difference compared to all other groups (*P* < 0.0001). The *G. mellonella* survival assay also revealed no significant differences in mortality between KP52, Δ*iroBCDN*, and Δ*iroBCDN-C*, with all three strains causing approximately 90% larval death within 36 h post-infection (Fig. 5D). The survival curve of NTUH-K2044 was similar, while ATCC 700603 induced a significantly lower mortality. No deaths were observed in the PBS control group. These results indicate that the deletion of the *iroBCDN* gene cluster does not significantly impact the virulence of ST11 CR-hvKP.

### RNA-seq analysis reveals gene expression changes and pathways affected by *iroBCDN* deletion

**Fig 6:**
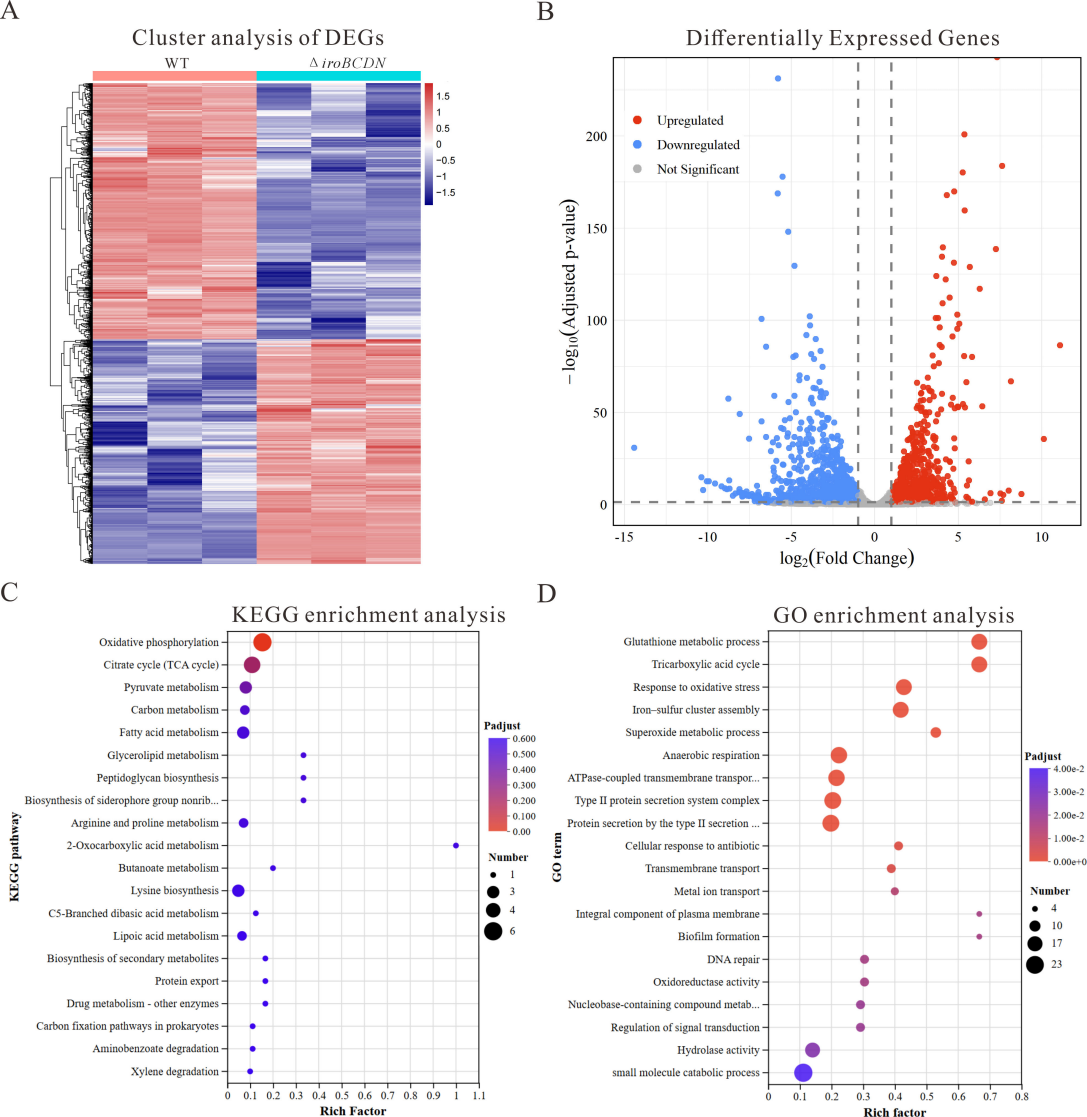
Transcriptomic characterization of wild-type KP52 and ∆*iroBCDN* mutant. (**A**) Hierarchical clustering heatmap based on transcriptomic profiles of wild-type KP52 and ∆*iroBCDN*. (**B**) Volcano plot showing differentially expressed genes (DEGs) between the two strains. Upregulated genes are shown in red, while downregulated genes are shown in blue. (**C**) KEGG pathway enrichment analysis of DEGs. (**D**) GO enrichment analysis of DEGs.

To investigate the global transcriptional effects of *iroBCDN* gene cluster deletion in ST11 CR-hvKP, we performed whole-transcriptome sequencing on the wild-type strain KP52 and the ∆*iroBCDN* mutant. The hierarchical clustering analysis demonstrated clear differences in gene expression profiles between the two strains (Fig. 6A). A total of 1,803 differentially expressed genes (DEGs) were identified in ∆*iroBCDN*, of which 842 were significantly upregulated, and 961 were downregulated (Fig. 6B). The upregulated genes included those involved in antioxidant defense mechanisms, such as *ahpC*, *ahpF*, *dps*, and *trxA*, which play crucial roles in mitigating oxidative stress and protecting the bacteria from cellular damage (Table 1). Additionally, genes associated with capsule biosynthesis, including *galE*, *rcsA*, and *rcsF*, were also upregulated, suggesting a potential increase in the production of the bacterial capsule, which is key for immune evasion and virulence. qRT-PCR validated the differential expression of *ahpC*, *ahpF*, *dps*, *trxA*, *galE*, *rcsA*, and *rcsF*, consistent with the RNA-seq results (Fig. S1). Furthermore, genes involved in energy metabolism, such as *focA*, *eno*, and *pykF*, were upregulated, reflecting an enhanced capacity for energy production likely to support the bacterial adaptation to the altered environmental conditions caused by the *iroBCDN* gene cluster deletion. Conversely, the downregulated genes primarily involved those linked to fimbrial synthesis, particularly *fimI*, which is essential for the formation of type 1 fimbriae, suggesting a reduced ability of the bacteria to adhere to host surfaces. Additionally, genes involved in carbon metabolism, including *malK* and *srlA*, were downregulated, pointing to a possible shift in the bacterium’s metabolic pathways and possibly affecting its ability to utilize certain carbon sources effectively. The KEGG pathway enrichment analysis revealed that DEGs were primarily enriched in pathways related to oxidative phosphorylation, tricarboxylic acid (TCA) cycle, pyruvate metabolism, carbon metabolism, fatty acid metabolism, and glycerophospholipid metabolism (Fig. 6C). The gene ontology (GO) enrichment analysis further showed that the DEGs were involved in biological processes, such as glutathione metabolism, oxidative stress response, iron-sulfur cluster assembly, anaerobic respiration, DNA repair, biofilm formation, and transmembrane transport (Fig. 6D).

**TABLE 1:** Transcriptomic analysis of differentially expressed genes

Gene name	Fold change	*P* value	*P* adj	Gene description
Antioxidant-related genes				
*ahpC*	4.78294762	1.05E−32	2.74E−31	Allyl hydroperoxide reductase, Ahp component
*trxA*	3.09729621	2.33E−09	1.83E−08	Thioredoxin
*dps*	2.77782498	1.84E−04	7.95E−04	DNA protection during starvation protein
*ahpF*	2.73067601	3.82E−14	4.24E−13	Allyl hydroperoxide reductase, Ahp component
Capsule biosynthesis genes				
*galE*	2.04790216	5.91E−05	2.79E−04	UDP-glucose 4-epimerase
*rcsA*	1.18711816	9.91E−09	7.32E−08	Transcriptional activator for capsule biosynthesis
*rcsF*	1.82359499	1.77E−03	6.31E−03	Outer membrane lipoprotein
Energy metabolism genes				
*focA*	3.79158398	1.50E−11	1.43E−10	Formate channel FocA
*eno*	3.55222705	8.28E−15	9.70E−14	Phosphopyruvate hydratase
*pykF*	2.09629041	1.35E−10	1.19E−09	Pyruvate kinase
Fimbrial synthesis-related genes				
*fimI*	−7.90609500	5.94E−06	3.26E−05	Type 1 fimbrial protein subunit FimI
Carbon metabolism genes				
*malK*	−6.51426318	8.94E−07	5.48E−06	Maltose/maltodextrin transporter ATP-binding protein
*srlA*	−4.54084659	5.05E−03	1.59E−02	Glucitol/sorbitol-specific PTS enzyme IIC component

### The deletion of *iroBCDN* enhances the fitness and survival of ST11 CR-hvKP

**Fig 7:**
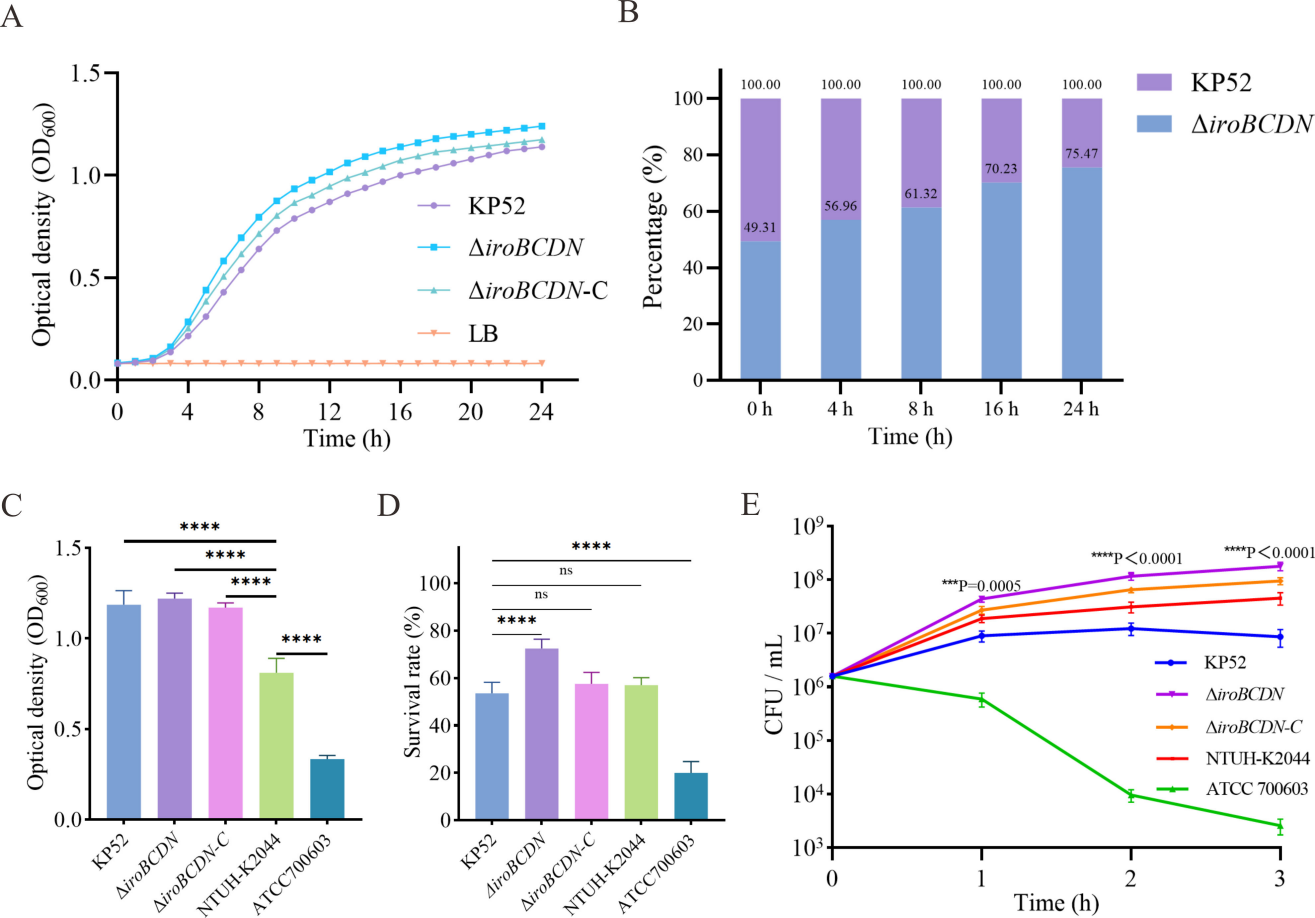
Phenotypic characterization of the ∆*iroBCDN* mutant in ST11 CR-hvKP. (**A**) Growth curves of the wild-type KP52, the ∆*iroBCDN* mutant, and the complemented strain ∆*iroBCDN*-C in LB medium. (**B**) *In vitro* competition assay between KP52 and the ∆*iroBCDN* mutant during co-culture. The proportion of the mutant strain increased over 24 h. (**C**) Quantification of the biofilm formation by crystal violet staining and (**D**) H_2_O_2_ killing assay. Statistical significance was determined using one-way ANOVA with multiple comparisons. Statistical annotations are indicated as follows: ns, not significant; ****P < 0.0001. (**E**) Whole-blood survival assay. Statistical significance was determined at each time point using unpaired two-tailed *t*-tests to compare ∆*iroBCDN* and KP52. *P*-values shown in the figure correspond to these pairwise comparisons: ***P < 0.001 and ****P < 0.0001. “ns” indicates not significant (*P* ≥ 0.05).

We further investigated the effect of *iroBCDN* deletion on bacterial fitness and survival. The growth curve analysis revealed that the ∆*iroBCDN* strain exhibited a significantly faster growth rate than the wild-type strain KP52, suggesting a potential fitness advantage conferred by the deletion of the *iroBCDN* gene cluster. In contrast, the complemented strain (∆*iroBCDN-C*) displayed a slower growth rate compared to the mutant strain under the same conditions (Fig. 7A). These results imply that reintroduction of *iroBCDN* may impose a metabolic burden or regulatory constraint that reduces the overall growth efficiency. In line with the growth advantage, competition assays demonstrated that the ∆*iroBCDN* strain progressively outcompeted the wild-type strain during co-culture, with its relative proportion increasing over time (Fig. 7B). The relative fitness of the ∆*iroBCDN* mutant compared to the wild-type strain KP52 was calculated to be approximately 1.86, indicating that the mutant strain exhibits a significant competitive advantage during co-culture under the tested conditions.

Despite the observed upregulation of antioxidant-related genes in the ∆*iroBCDN* mutant, biofilm formation assays revealed no significant difference between the mutant and wild-type KP52 (Fig. 7C). This suggests that the increased expression of these genes does not lead to enhanced biofilm production. In the H_2_O_2_ killing assay, the ∆*iroBCDN* mutant showed a survival advantage relative to KP52 (Fig. 7D). Consistently, in whole blood survival assays, however, the ∆*iroBCDN* strain exhibited significantly enhanced survival compared to the wild-type strain (Fig. 7E). Taken together, these findings suggest that deletion of *iroBCDN* promotes bacterial fitness and host survival potential.

## DISCUSSION

In this study, isolates were classified as CR-hvKP on the basis of carrying multiple key virulence loci (*rmpA*, *rmpA2*, *iucA*, *iroB*, or *peg-344*) and attaining virulence scores ≥ 3 in Kleborate analysis. These genomic features were corroborated by *Galleria mellonella* infection assays, which consistently demonstrated higher virulence in these isolates compared with CRKP controls. Among the 41 ST11 CR-hvKP strains, only two ST11-KL47 strains carried the *iroBCDN* gene cluster, while the others lacked this virulence gene cluster. This observation suggests that the *iroBCDN* absence is a common feature of epidemic ST11 CR-hvKP strains. Previous studies have observed that ST11-KL64 and ST11-KL47 belong to a major evolutionary branch, with ST11-KL47 being the ancestral strain of ST11-KL64 (30, 31). Data indicate that the evolution from KL47 to KL64 is becoming the dominant trend, with the previously dominant subclone ST11-KL47 being gradually replaced by ST11-KL64 in the CRKP ST11 lineage since 2015 (32, 33). A recent investigation has demonstrated that the ST11-KL64 CR-HvKP has emerged as a dominant pathogen of hospital infections in western China (34). Although *iroBCDN* loss is frequently observed in KL64 genomes, our current data do not establish whether this genetic change directly contributed to the clonal replacement. Larger-scale phylogenomic analyses will be required to clarify any causal relationships.

The *iroBCDN* gene cluster encoding the siderophore salmochelin is widely regarded as an important virulence determinant of HvKP (35). However, in prevalent ST11 CR-hvKP strains, this cluster is frequently absent (20). Contrary to expectation, the deletion of iroBCDN does not diminish the epidemic potential of these strains and may even confer fitness advantages under specific conditions. Instead, the frequent absence of *iroBCDN* in ST11 CR-hvKP may be associated with enhanced adaptability in certain environments. In an *iroBCDN*-carrying ST11-KL47 strain (KP52), knockout mutants showed no reduction in virulence, as assessed by LDH release and *Galleria mellonella* infection, compared with wild-type and complemented strains. This unexpected finding suggests that the loss of *iroBCDN* does not necessarily attenuate the ST11 CR-hvKP virulence. One possible explanation is that ST11 CR-hvKP compensates for the loss of *iroBCDN* through other virulence genes. For example, after the deletion of *iroBCDN*, the expression of capsule biosynthesis-related genes increased, thus maintaining the strain’s high virulence. Furthermore, *Galleria mellonella* infection experiments confirmed that, even without *iroBCDN*, CR-hvKP still exhibited high virulence, suggesting that the loss of *iroBCDN* does not impair the pathogenicity of ST11 CR-hvKP.

Transcriptomic analysis revealed significant changes in the expression of 1803 genes in ST11 CR-hvKP after *iroBCDN* deletion. Among these, antioxidant genes (such as *ahpC *and *dps*) were upregulated, potentially enhancing the bacterium’s ability to cope with host oxidative stress (36, 37); capsule biosynthesis genes (such as *gale*, *rcsA*, and *rcsF*) were upregulated, potentially promoting capsule synthesis and enhancing resistance to host immune clearance (38, 39); and energy metabolism genes (such as *focA*, *eno*, and *pykF*) were upregulated, suggesting that the strain may optimize energy metabolism pathways to improve survival in nutrient-limited environments (40–42). In contrast, fimbrial synthesis genes (*fimI*) and carbon metabolism genes (*malK*, *srlA*) were downregulated, which may reduce fimbrial synthesis to decrease host recognition risk or adapt to different environmental conditions by adjusting carbon metabolism (43–45). The KEGG and GO pathway enrichment analyses further showed that the differentially expressed genes were primarily enriched in key metabolic pathways, such as oxidative phosphorylation, tricarboxylic acid cycle, carbon metabolism, fatty acid metabolism, and glycerophospholipid metabolism, as well as physiological processes, such as oxidative stress response and biofilm formation. This suggests that *iroBCDN* deletion may be associated with remodeling of the bacterial metabolic network and stress response mechanisms, providing a competitive advantage for the strain in complex ecological niches, such as hospital environments.

Based on the transcriptomic results, we validated the strain’s adaptability using growth curves, competition experiments, biofilm formation assays, and whole blood survival assays. The results showed that the *iroBCDN* knockout strain had a faster growth rate compared to the wild-type strain, and the growth rate of the *iroBCDN*-complemented strain was slower. Competition experiments also showed that the knockout strain had a growth advantage over the wild-type strain. Whole blood killing assays indicated that the *iroBCDN* knockout strain had enhanced resistance to serum killing. The upregulation of antioxidant and capsule biosynthesis genes in the knockout strain may explain this enhanced resistance, suggesting that the strain’s improved antioxidant capacity and immune evasion mechanisms contributed to its survival advantage.

However, this study has certain limitations. First, all functional assays were performed in a single *iroBCDN*-positive ST11-KL47 isolate. The scarcity of *iroBCDN*-positive ST11 isolates limited the feasibility of testing additional genetic backgrounds within this study. Second, the specific molecular mechanisms by which *iroBCDN* deletion regulates downstream gene expression remain unclear, and further research is needed to explore the regulatory network. Third, our virulence assessment was limited to cytotoxicity and *G. mellonella* infection models, which do not fully capture mammalian-specific host-pathogen interactions, such as lipocalin-2-mediated immune evasion (46). Thus, the impact of *iroBCDN* deletion on virulence in mammalian hosts requires further validation in appropriate animal models.

In summary, the deletion of *iroBCDN* enhances the strain’s ability to resist host defenses by upregulating antioxidant and capsule biosynthesis-related genes. It also increases survival and reproductive ability in different environments by optimizing energy metabolism and adjusting carbon metabolism pathways, which may provide ST11 CR-hvKP with ecological advantages that could facilitate its persistence.

## Data Availability

The Illumina sequencing data of 46 CR-hvKP isolates have been deposited in the NCBI database under BioProject accession number PRJNA1304229. The RNA-Seq data in this study can be available in the NCBI database under BioProject accession number PRJNA1304230.

## SUPPLEMENTAL MATERIAL

Figure S1 spectrum.02479-25-s0001.docx Validation of differentially expressed genes by qPCR.

Table S1 spectrum.02479-25-s0002.xlsx Species identification of 217 carbapenem‑resistant isolates by MALDI‑TOF MS.

Table S2 spectrum.02479-25-s0003.xlsx Primers used in this study.

Table S3 spectrum.02479-25-s0004.xlsx MICs of carbapenems for 46 carbapenem-resistant hypervirulent *Klebsiella pneumoniae*.

ASM does not own the copyrights to Supplemental Material that may be linked to, or accessed through, an article. The authors have granted ASM a non-exclusive, world-wide license to publish the Supplemental Material files. Please contact the corresponding author directly for reuse.
