# Supplementary material for: Epidemiological and functional insights into iroBCDN loss in ST11 carbapenem-resistant hypervirulent Klebsiella pneumoniae
Source: Microbiol Spectr. 2025 Dec 15;14(2):e02479-25. doi: 10.1128/spectrum.02479-25 (PMC12889106; doi:10.1128/spectrum.02479-25)
Supplement: Figure S1 — Validation of differentially expressed genes by qPCR. [file spectrum.02479-25-s0001.docx]

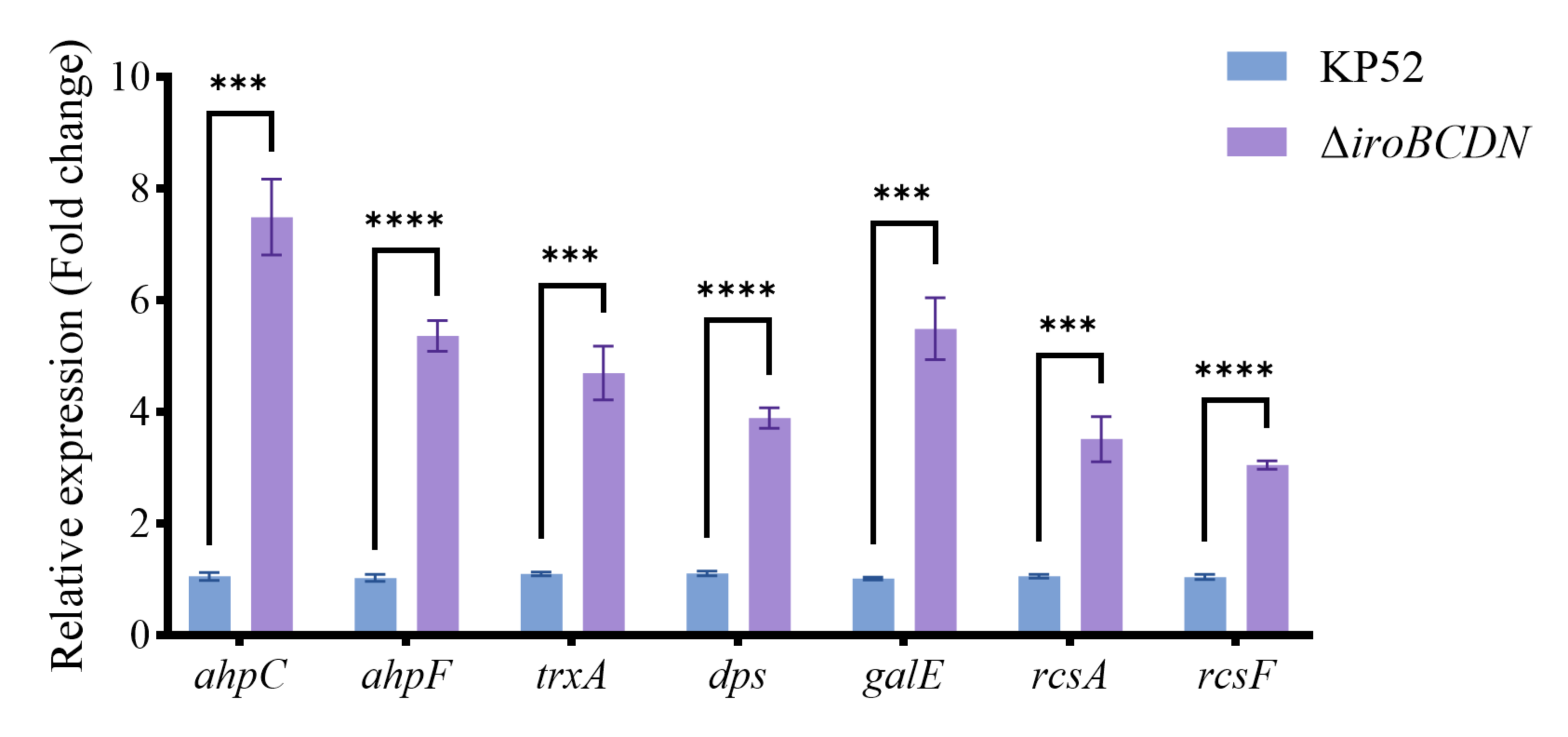


**Figure S1**. Validation of differentially expressed genes by qPCR. Statistical significance was determined by unpaired two-tailed Student’s t-test.
